# Man-in-the-barrel syndrome caused by cervical arachnoiditis in refractory coccidioidal meningitis

**DOI:** 10.1016/j.mmcr.2026.100818

**Published:** 2026-08-24

**Authors:** Sophia Marie Pelayo Tran, Tanya Savenka, Kwan L. Ng, George R. Thompson

**Affiliations:** a University of California Davis Medical School, Sacramento, CA, USA; b Division of Infectious Diseases, Department of Medicine, University of California Davis, Medical Center, Sacramento, CA, USA; c Department of Neurology, University of California Davis Medical Center, Sacramento, CA, USA; d Department of Medical Microbiology and Immunology, University of California Davis Medical, Center, Davis, CA, USA

**Keywords:** Coccidioidal meningitis, Man-in-a-barrel syndrome, Arachnoiditis, Spinal cord compression, Intrathecal amphotericin

## Abstract

We report a case of refractory coccidioidal meningitis presenting with bilateral upper-extremity weakness in a man-in-a-barrel distribution caused by cervical arachnoiditis, adhesive bands, and spinal cord compression. The patient was treated sequentially with fluconazole, other azole therapy, liposomal amphotericin B, intrathecal amphotericin B, and olorofim, with only partial disease control. Neuroimaging demonstrated progressive cervical leptomeningeal inflammation, cord tethering, and CT myelography illustrated adhesive arachnoiditis. Surgical lysis of adhesions and placement of a spinal Ommaya reservoir for intrathecal amphotericin delivery above and below the obstructed segment resulted in partial neurologic improvement. This case broadens the differential diagnosis of man-in-a-barrel syndrome to include active fungal meningitis with compressive cervical myelopathy and highlights the role of combined surgical-medical management in complicated fungal arachnoiditis.

## 1 Introduction

Coccidioidomycosis, or “Valley Fever”, is an invasive fungal disease caused by *C. immitis* or *C. posadasii*. This soil-dwelling organism is predominantly found in soil within the Southwestern United States, although it also exists in the state of Washington, Utah, and parts of Mexico and Central and South America [1]. Following inhalation of airborne arthroconidia (e.g., spores), respiratory infection is the most frequent clinical presentation [2]. In a minority of cases severe and/or disseminated infection may develop even in immunocompetent patients. The treatment of these forms of disease is challenging, and refractory or progressive disease may occur even in the setting of appropriate antifungal treatment [3,4].

The causal mechanisms of treatment failure have not been fully elucidated, but pharmacokinetic/pharmacodynamic factors, an inappropriate or aberrant host immune response, and pathogen specific traits have been proposed as contributory [5,6]. During infection of the central nervous system (CNS) *Coccidioides*, in some cases, induces a highly inflammatory and/or fibrotic response that may result in hydrocephalus or arachnoiditis [3].

We present a case of central nervous system (CNS) coccidioidomycosis with resultant arachnoiditis and upper-extremity weakness in a “man-in-a-barrel” distribution. Prolonged meningeal inflammation due to recalcitrant disease led to the development of arachnoid web formation causing spinal cord compression. Management required a multidisciplinary medical-surgical approach to improve the patient's symptoms and regain control of the infection. We review recent literature of causes of man-in-a-barrel syndrome with a focus on infectious causes of this condition and illustrate the complex approach required in the treatment of fungal arachnoiditis.

### 1.1 Case

A 49-year-old man presenting with 2 months of progressive fever and headache, decreased mental acuity, and ear pressure, was first diagnosed with coccidioidal meningitis on Day 0 with cerebrospinal fluid (CSF) pleocytosis (397 cells/μL), an elevated protein (183 mg/dL) and positive coccidioidal complement fixation antibodies (1:32). His initial evaluation also included a negative meningitis/encephalitis panel (Biofire Filmarray, Salt Lake City, UT, USA) and negative cultures. A non-contrast head CT at this time was normal, while a contrasted MRI study showed mild enhancement of the basilar artery suggesting potential basilar meningitis. He was initially treated with fluconazole 600mg/day, however the resulting polyarthralgias necessitated a change to itraconazole capsules 200mg twice daily. Despite full compliance with his prescribed antifungal on approximately Day 1,460, he returned with concerns of treatment failure, with worsening headaches, and worsening leptomeningeal disease found on magnetic resonance imaging (MRI). He was treated with intravenous liposomal amphotericin B (L-AMB) 5mg/kg until clinical improvement on day 1480 and was then transitioned to oral posaconazole 300mg orally daily. On approximately Day 1,520, after 40 days of treatment with posaconazole and serum drug levels confirmed therapeutic (2.83 μg/mL), he developed new left facial numbness; CSF protein was again elevated and a repeat MRI showed skull-base leptomeningeal nodularity and cerebellar edema along the trigeminal V3 branch. He received high-dose fluconazole (1000mg daily) and a fourteen-day prednisone taper (beginning with 80mg daily) with improvement in cerebrospinal fluid parameters (pleocytosis and CSF protein decreased).

On approximately Day 1,580, the patient returned with worsening circumferential headaches and worsening of his CSF pleocytosis (444 cells/μL), elevated CSF protein (301 mg/dL), and new cervical spinal involvement with cord tethering. He was treated with dexamethasone 20mg/day, intravenous L-AMB 5mg/kg, and high-dose fluconazole 800 mg twice daily. Due to the refractory nature of his infection he was transferred to our center for evaluation and management. During this hospitalization, at approximately Day 1585 the patient was enrolled in a phase IIb trial of oral olorofim (90 mg orally twice daily) specifically designed for patients with invasive fungal disease resistant, or refractory to standard therapy, or with intolerance to alternative antifungal agents [7]. He was concurrently investigated for any underlying immunodeficiency, although no clear etiology contributing to disseminated coccidioidomycosis was identified. Following enrollment the patient's symptoms resolved, his CSF and coccidioidal serologies improved (1:2), and he was observed through approximately Day 1,790, representing seven months of objective improvement.

Unfortunately, at approximately Day 1,790, he presented again with recurrence of his headache and fatigue, and on admission CSF studies showed pleocytosis with CSF WBC 24 cells/μL, a glucose of 40 mg/dL, and a markedly elevated CSF protein of 4011 mg/dL. His CSF coccidioidal complement fixation titer had increased to 1:32. Due to the lack of alternative therapeutic options, olorofim was continued, and L-AMB 5 mg/kg daily and voriconazole 400mg orally twice daily prescribed. Intrathecal amphotericin B deoxycholate was also started at approximately Day 1792 and given via an implanted Ommaya reservoir at a dose of 0.4 mg per day.

**Fig. 1:**
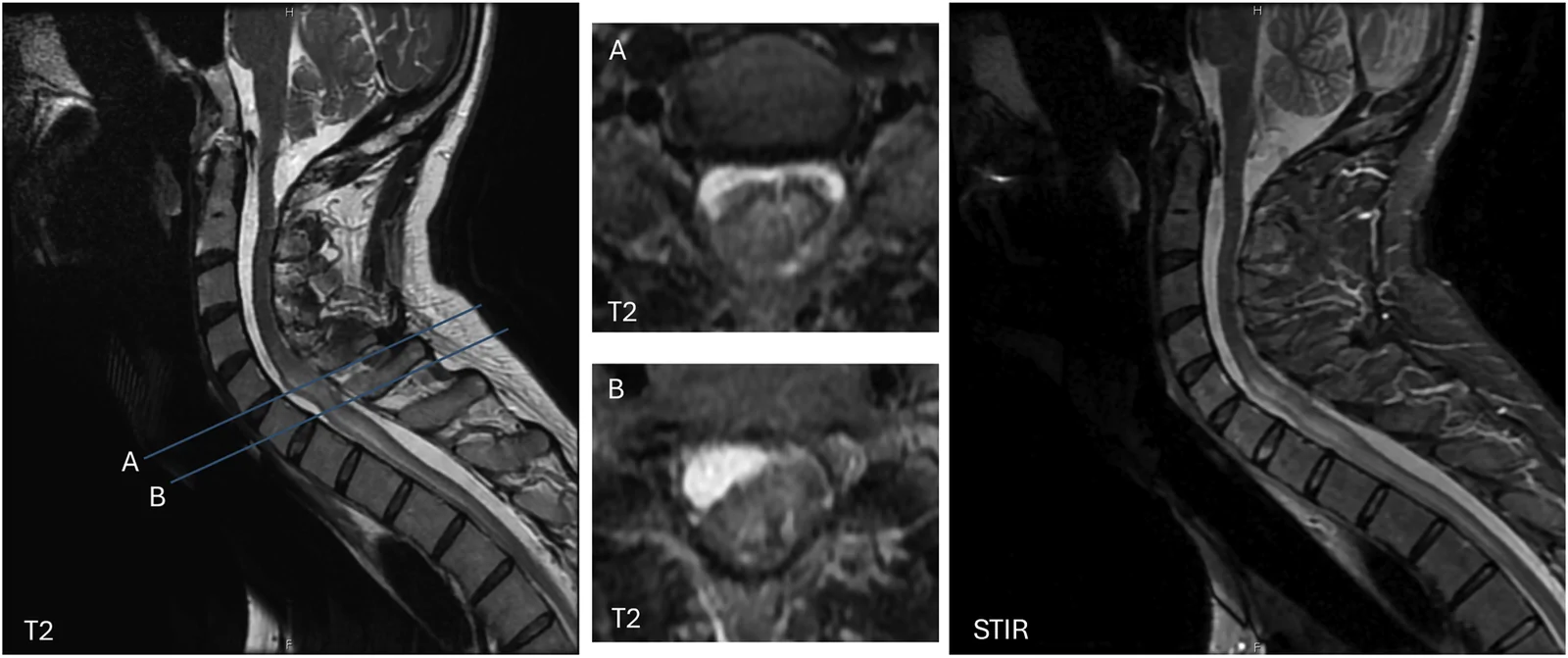
Cervical Spine with cord expansion and myelopathic changes with adhesion band causing focal adhesion of spinal cord from C4 to T1. (Left) T2 Sagittal; (Middle) T2 Axial cross-section A and B (Right) STIR Sagittal.

At approximately Day 1,811, after three weeks of intrathecal amphotericin B therapy, the patient developed bilateral upper extremity weakness. A CT myelogram was performed due to concern of arachnoiditis with additional concerns for potential compartmentalization of the CSF space. C4-T1 post-infectious intrathecal adhesive bands were observed with corresponding signal abnormalities and arachnoiditis (Fig. 1). Olorofim was stopped on day 1,811, while voriconazole and liposomal amphotericin B were continued. Over the subsequent seven days, through approximately Day 1,818, his physical exam progressed with development of nystagmus, bilateral upper and lower extremity paresthesia without sensory loss, and new proximal (4/5) and distal (3/5) bilateral upper-extremity weakness with intact strength of the lower-extremities. The selective involvement of the upper extremities with preserved lower-extremity strength and intact sensation was consistent with man-in-a-barrel syndrome.

**Fig. 2:**
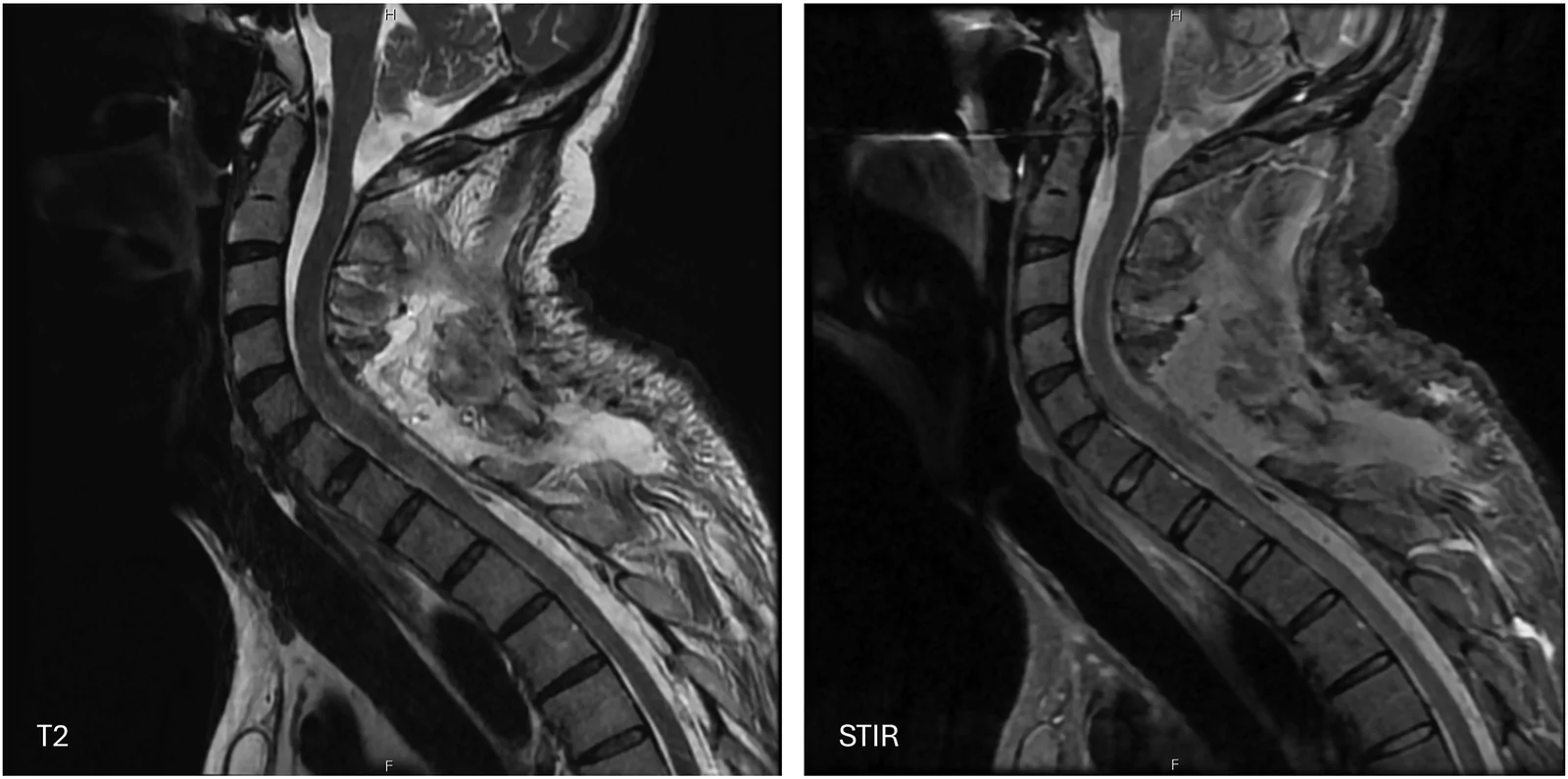
Six weeks post-operative Cervical Spine with resolution of cord expansion and myelopathic changes.

Neurology evaluation noted the “man-in-a-barrel” pattern and attributed this to impaired CSF flow from C6–C7 arachnoid webs and meningeal inflammation causing distal cervical cord compression. The patient underwent surgical lysis of cervical spine adhesions and placement of a T1 level spinal Ommaya reservoir with a “T-piece” to ensure that intrathecal therapy could be delivered above and below the site of arachnoiditis and surgical lysis. The IT dose of AmB was not escalated given the concerns that amphotericin B may be contributing to arachnoiditis, however no other effective treatment options were available for his refractory disease. Post-operatively there was noted partial improvement in his bilateral upper extremity weakness, and by approximately Day 1,860, six weeks after surgery, his cervical spine imaging showed resolution of cord expansion and myelopathic changes (Fig. 2). Due to improvement in symptoms and CSF parameters on day 1863 his liposomal amphotericin B was stopped. By approximately Day 1,970, after six months of intrathecal amphotericin treatment and normalization of his CSF parameters, with increasing AST and ALT elevation, he was transitioned from intrathecal therapy and voriconazole to oral isavuconazole (200mg daily following oral loading) alone. At approximately Day 4,160, after six years of continuous isavuconazole therapy, he remains on treatment with no evidence of recurrent disease (normal CSF cell count, protein, glucose and undetectable coccidioidal antibody titer). His neurologic exam remains stable.

## 2 Discussion

The management of progressive and refractory coccidioidal meningitis is difficult and every attempt should be made to optimize pharmacokinetic/pharmacodynamic parameters. Fluconazole and voriconazole exhibit CSF levels ∼50% of their serum levels, while isavuconazole CSF levels are ∼10-30% of serum drug levels. Posaconazole and itraconazole have very poor CSF penetration, however animal models and patient cohort studies have shown efficacy in the treatment of CNS coccidioidomycosis [3,4,7,8]. Following fluconazole failure, our center generally prefers voriconazole or isavuconazole, with other triazoles relegated to refractory or intolerance to first and second-line agents. Newer agents, including olorofim and fosmanogepix, may play a large role in the treatment algorithm for refractory disease pending additional clinical experience [9,10]. Therapeutic drug monitoring is also essential to ensure compliance and appropriate drug levels in an attempt to optimize efficacy [5].

Coccidioidal meningitis is the most common manifestation of central nervous system coccidioidomycosis, and despite appropriate therapy hydrocephalus, brain abscess, vasculitis, spinal arachnoiditis, and/or syrinx may develop [11]. Spinal arachnoiditis results from inflammation and scarring of the arachnoid mater, leading to adhesions, nerve root clumping, and impaired CSF circulation due to fibrin and collagen deposition. It occurs in approximately 10% of patients with coccidioidal meningitis and may present with back pain, extremity weakness, or paresis [12]. In our patient, severe spinal arachnoiditis and adhesive bands produced cervical cord tethering and compression, resulting in the characteristic man-in-a-barrel pattern of bilateral upper-extremity weakness.

Man-in-a-barrel syndrome (MIBS) is a rare neurological condition caused by isolated brachial diplegia with normal sensation in the upper extremities and preserved motor function in the lower extremities that makes it appear as if the patient is “stuck in a barrel”. The syndrome was first described by Mohr in 1969, and subsequently named “man-in-a-barrel syndrome” based on its classical appearance [13]. MIBS can arise from lesions at multiple levels of the neuroaxis, and its differential diagnosis is broad [14]. The most common reported mechanism is watershed infarction in the border zones between the anterior and middle cerebral artery territories, typically in the setting of profound hypotension [15,16]. However, MIBS has also been reported with intracranial metastases, cervical spinal pathology causing ischemia or compression, bilateral brachial plexopathy, and a variety of autoimmune or inflammatory disorders affecting the peripheral nervous system [[17], [18], [19], [20], [21], [22]].

Infectious etiologies of MIBS are uncommon and to our knowledge coccidioidal meningitis has not previously been reported as a cause of man-in-a-barrel syndrome. Other infectious etiologies include epidural spinal abscess, in which both compressive and ischemic mechanisms are thought to contribute [23]. A prior case report described MIBS in the setting of disseminated pneumococcal infection, where MRI showed acute infarcts and angiography demonstrated vasculitis as the likely cause [24]. These reports underscore that infection-related MIBS may arise through vascular, compressive, or inflammatory mechanisms.

Only one published case, to our knowledge, has linked MIBS to infectious adhesive arachnoiditis. Karschnia et al. reported a patient who developed slowly progressive upper-extremity weakness two years after severe *Listeria monocytogenes* meningitis. In that case, lumbar puncture showed no evidence of active infection, MRI demonstrated sequestered CSF pockets in the cervical and thoracic spine, and CT myelography confirmed impaired contrast flow consistent with adhesive arachnoiditis [25]. Our patient differed in that the syndrome developed during active, refractory fungal meningitis with ongoing inflammation, progressive adhesive disease, and cervical cord compression rather than as a delayed postinfectious sequela. However, in addition to ongoing fungal replication within the CSF space, intrathecal amphotericin B may have additionally contributed to the development of MIBS in our case given the well-known inflammatory effects of IT-AmB [26].

This case highlights several teaching points. First, tenants of management in coccidioidal meningitis include optimization of PK/PD parameters to improve potential outcomes. Second, MIBS should not be attributed solely to watershed infarction; active CNS infection, cervical arachnoiditis, and irritants such as IT-amphotericin B are important alternative contributors. Third, CT myelography can be particularly helpful when MRI suggests arachnoiditis, CSF compartmentalization, or impaired flow, and fourth, refractory coccidioidal meningitis may require a combined medical-surgical approach, including shunting, intrathecal antifungal therapy, and lysis of adhesions, to relieve neurologic compromise and improve disease control. Recognition of meningitis-associated MIBS may therefore facilitate earlier diagnosis of this syndrome, prompt imaging of the brain and cervical spine, and timely intervention to preserve neurologic function.

## CRediT authorship contribution statement

**Sophia Marie Pelayo Tran:** Writing – original draft. **Tanya Savenka:** Writing – review & editing. **Kwan L. Ng:** Writing – review & editing. **George R. Thompson:** Conceptualization, Investigation, Project administration, Resources, Supervision, Writing – original draft.

## Authorship

All authors made substantial contributions in: (1) the conception and design of the report, acquisition of data, and interpretation of data, (2) drafting of the article and revising it critically for important intellectual content, and (3) final approval of the submitted version.

## Funding

None.

## Conflicts of interest

None.
